# Supplementary Low-Intensity Aerobic Training Improves Aerobic Capacity and Does Not Affect Psychomotor Performance in Professional Female Ballet Dancers

**DOI:** 10.2478/v10078-012-0008-6

**Published:** 2012-04-03

**Authors:** Ewelina Smol, Artur Fredyk

**Affiliations:** 1 Departament of Physiology, Academy of Physical Education, Katowice, Poland.; 2 Departament of Dance, Academy of Physical Education, Katowice, Poland.

**Keywords:** anaerobic threshold, aerobic training, ballet, maximal oxygen uptake

## Abstract

We investigated whether 6-week low-intensity aerobic training program used as a supplement to regular dance practice might improve both the aerobic capacity and psychomotor performance in female ballet dancers. To assess their maximal oxygen uptake (VO_2max_) and anaerobic threshold (AT), the dancers performed a standard graded bicycle ergometer exercise test until volitional exhaustion prior to and after the supplementary training. At both these occasions, the psychomotor performance (assessed as multiple choice reaction time) and number of correct responses to audio-visual stimuli was assessed at rest and immediately after cessation of maximal intensity exercise. The supplementary low-intensity exercise training increased VO_2max_ and markedly shifted AT toward higher absolute workload. Immediately after completion of the graded exercise to volitional exhaustion, the ballerinas’ psychomotor performance remained at the pre-exercise (resting) level. Neither the resting nor the maximal multiple choice reaction time and accuracy of responses were affected by the supplementary aerobic training. The results of this study indicate that addition of low-intensity aerobic training to regular dance practice increases aerobic capacity of ballerinas with no loss of speed and accuracy of their psychomotor reaction.

## Introduction

Classical ballet applying the five positions of the feet and the turnout of the legs can be classified as a high-intensity intermittent exercise mostly designed to improve movement technique (Wyon et al., 2004). Ballet training session (so-called lesson) consists of sections of low to moderate intensity (*adage*) and very short, high intensity exercise (*temps levels*) with intervals of several minutes, which involve both aerobic and anaerobic metabolism (Wyon et al., 2004; Wyon, 2005). Overall energy cost of a ballet dance exercise is very high. For example, in case of *tours piques* the anaerobic system was estimated to deliver 61–74% of energy needed for ATP resynthesis in working skeletal muscles (Guidetti et al., 2007). In the same study it was proved that the metabolic power for 30 s *tours piques* was 1.6 times larger than the subject’s VO_2max_ (Guidetti et al., 2007). Thus, professional classical dancer’s fitness development is as important as skill development in meeting the demands of contemporary choreography.

A significant part of muscular work in classical ballet, especially during warm-up sessions, is aerobic in nature (Schantz and Astrand, 1984; Cohen et al., 1982). Endurance capacity expressed as VO_2_max is significantly lower in classical ballet dancers compared to competitive athletes (Baldari and Guidetti, 2001; Koutedakis et al., 2004). Moreover, data collected by several research groups show that maximal oxygen uptake in dancers (either female or male) is comparable to untrained individuals (Baldari and Guidetti, 2001; Koutedakis et al., 2004; Wyon et al., 2007; Twitchett et al., 2009). Several researchers suggest that training based solely on dance does not provide adequate stimuli for the enhancement of cardiorespiratory functions (Baldari and Guidetti, 2001; Cohen et al., 1982; Wyon et al., 2004; 2005) and Koutedakis et al. (2004) suggest that workloads typically encountered during ballet lessons are too low to enhance aerobic capacity. Classical ballet dance training is mostly designed to develop movement technique and the sense of rhythm (Wyon et al., 2004; 2007; Koutedikas et al., 2004; Rafferty, 2010). Supplementing it with aerobic training might bring considerable benefits especially a reduction of central and/or peripheral fatigue (Hoffman et al., 1997).

Physically fit individuals compared to sedentary ones perform better in cognitive tasks (Tomporowski and Ellis, 1986; Etnier et al., 2006; Masley et al., 2009). There are reports showing that well-trained subjects have shorter psychomotor reaction time than sedentary ones both at rest and during exercise (Hansen et al., 2004; Chmura and Nazar, 2010). Moreover, the workload at optimal reaction time is higher in well-trained athletes than in untrained subjects, indicating positive effects of high intensity exercise on psychomotor performance (Chmura and Nazar, 2010; Kashihara et al., 2009). Endurance training-related improvement in psychomotor performance is associated with improved cardiopulmonary function (Etnier et al., 2006; Brisswalter et al., 2002).

Reports on the influence of supplementary training on maximal oxygen uptake in dancers are scarce. Wyon et al. (2007) did not find any significant correlation between the use of supplementary forms of regular physical activity (aerobic training, strength training, pilates) and maximal oxygen uptake values (VO_2_max) when assessing the fitness level in professional male and female dancers. In contrast, Koutedakis et al. (2007) showed that female modern dance students attained higher levels of VO_2_max and improvement in dance performance following supplementary aerobic training. However, there is no published study concerning this matter in classical ballet dancers. For this reason, we decided to examine whether adding a supplementary low intensity aerobic training program to regular dance practice would improve VO_2_max and psychomotor performance in classical ballet dancers.

## Material and Methods

### Subjects

Six professional female ballet dancers volunteered for the study. All the subjects started dancing at 9 years of age and were subjected to regular dance training for at least 12 years. During their work as members of the *corps de ballet* (including at least two years immediately preceding the study) they danced on the average about 6 times (a total of 24 h) per week. They had not been involved in other forms of regular physical activity. After being informed about the purpose of the study, all the subjects signed a written consent to participate in the study. The study protocol was approved by the Ethics Committee of the Academy of Physical Education in Katowice, Poland.

All the volunteers were clinically healthy and in good nutritional status, and their habitual diet was assessed with the use of a questionnaire. The dancers recorded their food intake over a 3-day period just before the commencement of exercise tests, and the daily records were analyzed for energy and macronutrients intake using a computer program Dietus (B.U.I. InFit 1995, Poland). Basic anthropometric characteristics of the subjects are presented in Table 1.

### Study design

The experimental protocol consisted of anthropometric measurements, a psychomotor performance test and graded exercise test for the evaluation of VO_2_max and anaerobic threshold (AT). All anthropometric measurements, the psychomotor performance test and exercise test were performed both prior to the beginning of aerobic training (pre-T) and following a 6-week supplementary aerobic training (post–T).

Body composition was assessed using bio-electrical impedance (Tanita body composition analyzer TBF-300). All subjects cycled on a 828 Monark (Sweden) ergometer with intensity increasing by 30 W every 3 min until volitional exhaustion. Minute ventilation (Ve) and oxygen uptake (VO_2_) were analyzed continuously (breath-by-breath) for 1 min at rest and at the third minute of each workload using standard technique of open-circuit spirometry (Yeager). Heart rate (HR) was recorded continuously using a PE 3000 Sport Tester (Polar Electro, Finland).

To determine the anaerobic threshold, fingertip capillary blood samples for lactate concentration assessment were taken at rest, at the third minute of each workload, and at the fifth minute of post-exercise recovery. Blood lactate concentration was measured by the standard enzymatic method using commercial kits (Boehringer-Mannheim, Germany) and a model UV-1201 UV/VIS Shimadzu spectrophotometer. The sensitivity of the blood lactate assay was 0.1 mmol/l. Additionally, fingertip capillary blood samples were taken both at rest and during maximal exercise for the assessment of acid-base balance parameters (pO_2_, pCO_2_, pH, actual and standard bicarbonate concentration [HCO_3_^−^], and actual and standard base excess, BE) using a model Rapid Lab 248 (Bayer Diagnostics) blood gas analyzer.

The anaerobic threshold (AT), defined as exercise workload at which the concentration of plasma lactate begins to increase in a nonlinear fashion, was calculated using the log-log method (log [La^−^] *vs*. log exercise intensity) with two segmental linear regressions according to Beaver et al. (1985).

### Psychomotor performance testing

Psychomotor performance was evaluated based on results of multiple choice reaction time and the number of correct responses to positive stimuli. The test consisted of reacting to a randomized sequence of 15 “real” (red light or a sound) and 15 “false” (green or yellow light) stimuli. The subjects were asked to press and release, as quickly as possible, a button on the right handlebar of the cycle ergometer in response to a red light, the button on the left handlebar in response to a sound, and not to react to false stimuli. The reaction time was measured with 0.01s accuracy using a reaction time measuring device (model MRK 433, Zabrze, Poland) and the results were given as a mean of 15 responses to real stimuli.

### Training protocol

The training program consisted of aerobic cycling exercise (using the 828 Monark, ergometer) 6 days/week, each session lasting 30 min, for successive 6 weeks. The applied workloads were determined based on HR that was kept at 90 beats/min during the first 2 weeks, at 100 beats/min during the next 2 weeks, and at 110 and 120 beats/min in the fifth and sixth week of training, respectively.

### Statistical analysis

All variables are reported as the mean ± S.D. Statistical significance of differences between means was assessed using a 2-way repeated measures ANOVA with training status (pre- or post-) and exercise status (rest or volitional exhaustion) as the repeated measures’ factors, followed by the Bonferroni test when appropriate, or by Student’s *t* test for paired variables. All statistical analyses were carried out using the statistical software package STATISTICA v. 6.0 (StatSoft Inc., Tulsa, OK, USA).

## Results

Body mass and body composition of the studied subjects before and after supplementary aerobic training are presented in Table 2. The training caused no significant change in any of these indices.

**Figure 1:**
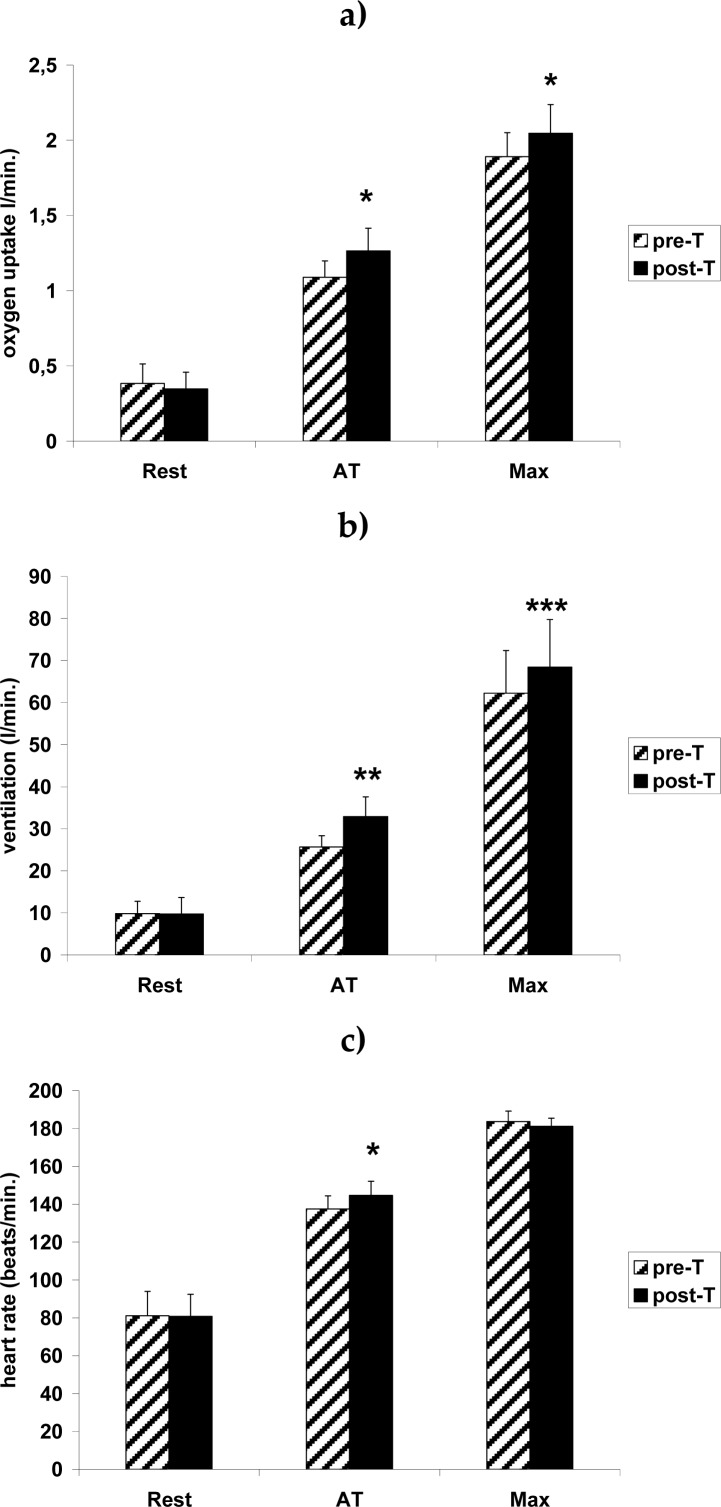
Oxygen uptake (a), minute ventilation (b) and minute heart rate (c) in female ballet dancers at rest (Rest), at anaerobic threshold intensity (AT) and at maximal intensity (Max) prior to (pre-T) and after (post-T) 6 weeks of aerobic training. All results expressed as the mean ± S.D. (n=6). Oxygen uptake 3-way ANOVA results: Training effect: F_1,5_=16.91, p=0.0092; Exercise effect: F_2,10_=681.32, p<10^−6^; Training × Exercise interaction effect: F_2,10_=6.56, p=0.015. Minute ventilation 3-way ANOVA results: Training effect: F_1,5_=90.57, p=0.00022; Exercise effect: F_2,10_=151.43, p<10^−6^; Training × Exercise interaction effect: F_2,10_=17.08, p=0.00060. Heart rate 3-way ANOVA results: Training effect: F_1,5_=1.27, p=0.31; Exercise effect: F_2,10_=240.64, p<10^−6^; Training × Exercise interaction effect: F_2,10_=8.54, p=0.0069. * - p < 0.05, ** - p < 0.01, *** - p < 0.001 vs. the respective pre-T value.

**Figure 2:**
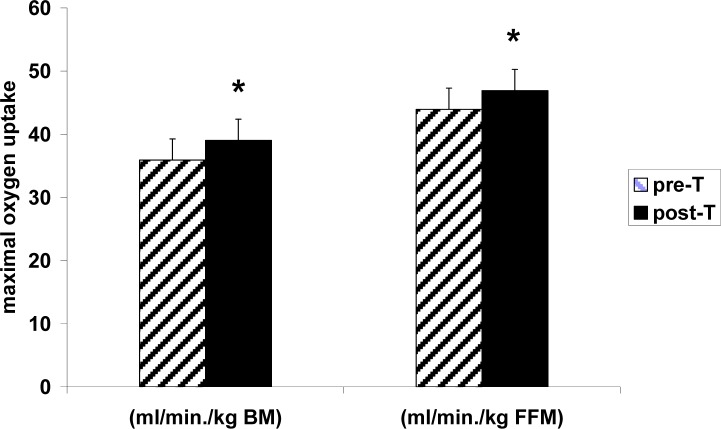
Maximal oxygen uptake per kilogram of body mass (BM) or kilogram of fat free mass (FFM) in female ballet dancers prior to (pre-T) and after 6 weeks of supplementary aerobic training (post-T). All results are expressed as the mean ± S.D. (n=6). * - p < 0.05 vs. the respective pre-T value; Student’s t test for paired variables.

Resting oxygen uptake (VO_2_), heart rate (HR) and minute ventilation (V_e_) (Figure 1a–c) did not show any deviation from normal values and were not significantly affected by the supplementary aerobic training. Maximal VO_2_ (both absolute and relative to body mass and FFM) and maximal V_e_ were significantly higher after the training (Figure 1a,b, Figure 2).

**Figure 3:**
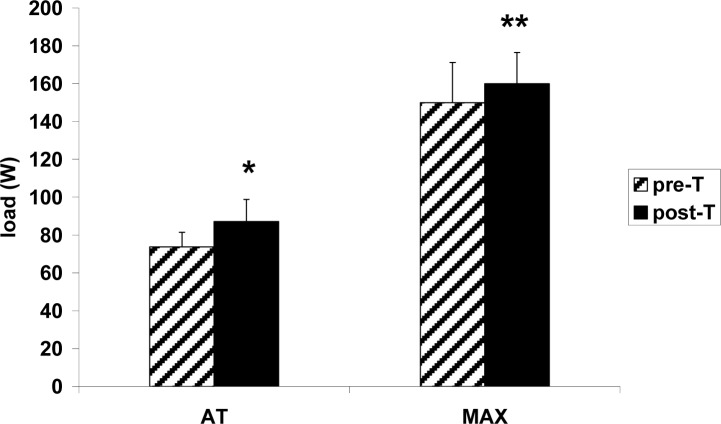
Anaerobic threshold workload (W) and maximal workload during incremental ergocycle exercise (3-min workloads increased by 30 W till volitional exhaustion) in female ballet dancers prior to (pre-T) and after (post-T) 6 weeks of supplementary aerobic training. All data are expressed as the mean ± S.D. (n=6). Two-way ANOVA results: Training effect: F_1,5_=14.56, p=0.012; Exercise effect: F_1,5_=461.32, p=4×10^−6^; Training × Exercise interaction effect: F_1,5_=2.55, p=0.17. * - p < 0.05, ** - p < 0.01 vs. the respective pre-T value.

**Figure 4:**
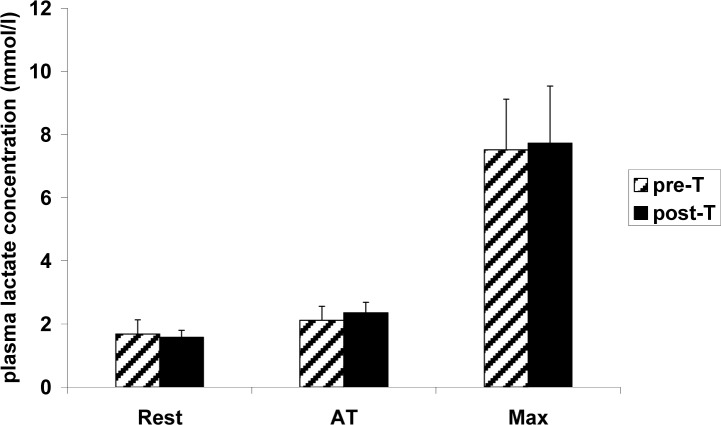
Plasma lactate concentration in female ballet dancers at rest, at anaerobic threshold intensity (AT), and at maximal intensity (Max) prior to (pre-T) and after (post-T) 6 weeks of supplementary aerobic training. All data are expressed as the mean ± S.D. (n=6). Three-way ANOVA results: Training effect: F_1,5_=1.81, p=0.24; Exercise effect: F_2,10_=120.29, p<10^−6^; Training × Exercise interaction effect: F_2,10_=0.87, p=0.45.

Anaerobic threshold (AT) prior to the supplementary aerobic training appeared at the workload of 73.8 W (Figure 3) and shifted to a significantly higher load (87.3 W) after the training. There was no significant difference in blood lactate concentration at AT prior to and after the training (Figure 3). There was no significant, supplementary training-related change in blood LA level either at rest, or at AT, or at maximal workload (Figure 4). Similarly, no significant, training-related change was observed in blood acid-base balance parameters, either at rest or after maximal exercise (Table 3).

Effects of supplementary aerobic training on mean, minimal and maximal choice reaction time at rest and immediately post-exercise are illustrated by the data summarized in Table 4.

There was no significant difference between the pre- and post-exercise reaction time either during the pre-training or post-training trials, and neither the resting, nor the post-exercise reaction time differed between the pre-training and post-training trials.

## Discussion

Pre-training VO_2_max tests revealed that the dancers showed low to moderate physical capacity, typical of untrained individuals, which is consistent with earlier reports (Wyon, 2005; Twitchett et al., 2009; Koutedakis et al., 1999; Koutedakis and Jamuratas, 2004). Afterwards, we subjected them to low intensity endurance training, with heart rate below 120 beats/min. The effectiveness of the training was evidenced by significant increases in both VO_2_max and AT. However, the VO_2_max in the study group remained below that reported previously (Wyon et al., 2007). This was despite the fact that the dancers were engaged in preparation for the new season, which should be associated with highest VO_2_max (Koutedakis et al., 1999), and that they showed similar body composition, education level and professional training to those described by other authors (Yannakoulia et al., 2000; Koutedakis and Jamurtas, 2004). This disagreement might be related in part to the use of an ergocycle and not a treadmill test that is known to give slightly higher VO_2_max values.

The relatively low AT level in our study group, which was within the range typical of untrained individuals, was in line with the VO_2_max findings discussed above. So far, the anaerobic threshold level in ballerinas (both adolescent and adult) has only been measured by non-invasive methods (Wyon et al., 2007; Baldari and Guidetti, 2001; Guidetti et al., 2008), and has only been compared between teenager ballerinas (13–16 years of age) and age-matched female gymnasts. The former showed AT levels similar to age-matched healthy sedentary girls and significantly lower than that in the gymnasts (Baldari and Guidetti, 2001).

These findings demonstrate that the length of professional practice in excess of 4–6 years does not affect aerobic capacity in female classical dancers.

In spite of a significant training-related increase in maximal workload there was no significant increase in blood lactate concentration at this workload. This indicates improved aerobic metabolism in the skeletal muscles. In line with this argument was the lack of significant training-related changes in the acid-base balance parameters. Altogether, these data demonstrate considerable benefit from the supplementary training despite VO_2_max remaining in the range typical of untrained healthy females. The effect of endurance training on psychomotor performance has not been previously studied in classical ballet dancers. Similar studies performed in athletes have shown a decrease after maximal exercise (Lemmink and Visscher, 2005), whereas some improvement was observed during exercises employing submaximal intensities (Chmura and Nazar, 2010; Chmura et al., 1994; 1998). Since classical ballet dancing involves mainly very high intensity effort, we believed that using submaximal intensity exercise would be pointless and hence we resorted to the use of maximal exercise. Resting reaction times found in professional female ballet dancers in this study were longer than those reported by other authors in male athletes (Chmura et al., 1994; 1998; Chmura and Nazar, 2010) in spite of the identical testing procedure. This disparity might be related both to the well-known differences in reaction time between males and females (Adam et al., 1999; Dogan, 2009) and to differences in task-oriented specific training protocols (Guizani et al., 2006a; Guizani et al., 2006b; Ryan et al., 2004). Endurance training was reported to decrease psychomotor performance after maximal exercise in male athletes (Chmura and Nazar, 2010). Despite its low intensity, the aerobic training significantly (as shown by significant Training × Exercise interaction effect) and beneficially modified some physiological indices of aerobic performance in the studied subjects. These results are in agreement both with the assumption that workloads typically used during classical ballet training are too low to enhance aerobic capacity (Baldari and Guidetti, 2001; Cohen et al., 1982; Wyon et al., 2004; 2005) and with the suggestion that the desired improvement can be achieved with supplemental low-intensity physical activity (Koutedakis et al., 2004). Importantly, the results of our study demonstrate also that low-intensity endurance training does not decrease (in absolute terms, i.e. as measured by reaction time) ballerinas’ psychomotor performance during maximal exercise. This suggests that psychomotor performance of classical ballet dancers actually benefits from endurance training, which may be due to some adaptive changes in the CNS, e.g. to a better tolerance of central fatigue (Langfort et al., 2006; Dwyer and Browning, 2000). Again, the difference in the effects of endurance training on this variable in ballerinas and athletes may be related to a number of factors, e.g. to gender and endurance training intensity, which is considerably higher in the latter.

The results of this study show that low-intensity endurance training is a suitable supplementary form of physical activity for increasing aerobic capacity in female classical ballet dancers. Notably, this type of training improves endurance without decreasing psychomotor performance during maximal exercise, which seems particularly important in this sport discipline.

## Figures and Tables

**Table 1:** Basic anthropometric characteristics of the studied subjects

Variables	Mean ± SD
Age (years)	27.5 ± 6.5
Body height (cm)	165.7 ± 6.7
Body mass (kg)	52.7 ± 5.6
BMI (kg/m^2^)	19.2 ± 1.8

Results shown are the mean ± SD (n=6).

**Table 2:** Body mass and composition of female ballet dancers prior to and after 6 weeks of supplementary aerobic training

Variable	Pre-training	Post-training
Body mass (kg)	52.65 ± 5.57	52.67 ± 6.17
BMI (kg/m^2^)	19.17 ± 1.83	19.15 ± 1.88
Fat (%)	17.97 ± 7.69	16.60 ± 8.05
Fat mass (kg)	9.83 ± 5.47	9.07 ± 5.39
Fat free mass (kg)	42.83 ± 2.42	43.60 ± 3.00

Results shown are the mean ± S.D. (n=6).

**Table 3:** Acid–base profiles of female ballet dancers at rest and immediately after maximal exercise intensity prior to and after 6 weeks of supplementary aerobic training

Variables	Exercise status	Aerobic training status		Two-way ANOVA results
		Pre-training	Post-training	
pH	Resting	7.40 ± 0.04	7.40 ± 0.01	Training: F_1,5_=1.95, p=0.22
	Maximal	7.30 ± 0.04	7.29 ± 0.04	**Exercise: F_1,5_=6.84, p=0.047**
				Interaction: F_1,5_=1.73, p=0.43

pCO_2_ (mm Hg)	Resting	38.48 ± 5.09	37.93 ± 2.72	Training: F_1,5_=0.88, p=0.39
	Maximal	36.75 ± 2.69	35.08 ± 3.20	Exercise: F_1,5_=1.16, p=0.33
				Interaction: F_1,5_=0.69, p=0.44

HCO_3_^−^ (mmol/l)	Resting	22.90 ± 2.44	22.98 ± 1.34	Training: F_1,5_=1.52, p=0.27
	Maximal	18.28 ± 2.02	16.58 ± 2.27	**Exercise: F_1,5_=27.00, p=0.0035**
				Interaction: F_1,5_=3.36, p=0.13

standard HCO_3_^−^ (mmol/l)	Resting	23.02 ± 1.82	23.22 ± 0.88	Training: F_1,5_=2.05, p=0.21
	Maximal	18.87 ± 1.69	17.37 ± 1.80	**Exercise: F_1,5_=51.51, p=0.00082**
				Interaction: F_1,5_=3.79, p=0.11

BE concentration mmol/l)	Resting	1.98 ± 2.48	1.83 ± 1.34	Training: F_1,5_=0.63, p=0.46
	Maximal	−8.47 ± 3.24	−10.05 ± 2.80	**Exercise: F_1,5_=49.24, p=0.00091**
				Interaction: F_1,5_=1.88, p=0.23

Standard BE concentration (mmol/l)	Resting	−1.65 ± 2.10	−1.45 ± 1.06	Training: F_1,5_=0.83, p=0.40
	Maximal	−7.68 ± 2.95	−9.02 ± 2.46	**Exercise: F_1,5_=37.69, p=0.0017**
				Interaction: F_1,5_=2.02, p=0.21

Results shown are the mean ± S.D. (n=6). Statically significant ANOVA results are shown in boldtype.

**Table 4:** Mean, minimal and maximal reaction time and number of correct responses to a positive stimuli during psychomotor test of female ballet dancers at rest and immediately after maximal exercise intensity prior to and after 6 weeks of supplementary aerobic training

Variables	Exercise status	Aerobic training status		Two-way ANOVA results
		Pre-training	Post-training	
Mean reaction time (ms)	Resting	447.0 ± 48.7	434.0 ± 50.4	Training: F_1,5_= 1.95, p=0.22
	Maximal	437.0 ± 44.8	407.0 ± 73.0	Exercise: F_1,5_= 6.84, p=0.47
				Interaction: F_1,5_= 0.73, p=0.43

Minimal reaction time (ms)	Resting	290.0 ± 94.2	300.0 ± 61.6	Training: F_1,5_= 0.0012, p=0.97
	Maximal	271.0 ± 77.6	260.0 ± 63.6	**Exercise: F_1,5_= 7.80, p=0.038**
				Interaction: F_1,5_= 0.45, p=0.53

Maximal reaction time (ms)	Resting	642.0 ± 100.3	603.0 ± 75.0	Training: F_1,5_= 0.35, p=0.58
	Maximal	643.0 ± 11.1	638.0 ± 19.8	Exercise: F_1,5_= 0.11, p=0.75
				Interaction: F_1,5_= 0.33, p=0.59

Number of correct responses	Resting	14.2 ± 1.2	13.8 ± 2.0	Training: F_1,5_=0.00, p=1.00
	Maximal	14.3 ± 1.2	14.7 ± 0.5	Exercise: F_1,5_=1.88, p=0.23
				Interaction: F_1,5_=0.69, p=0.44

Results shown are the mean ± S.D. (n=6). Statically significant ANOVA results are shown in boldtype.
